# DNA damage‐induced cellular senescence is regulated by 53BP1 accumulation in the nuclear foci and phase separation

**DOI:** 10.1111/cpr.13398

**Published:** 2023-01-15

**Authors:** Tsukasa Oda, Nanami Gotoh, Tetsuhiro Kasamatsu, Hiroshi Handa, Takayuki Saitoh, Nobuo Sasaki

**Affiliations:** ^1^ Laboratory of Mucosal Ecosystem Design The Institute for Molecular and Cellular Regulation, Gunma University Maebashi Gunma Japan; ^2^ Graduate School of Health Sciences Gunma University Maebashi Gunma Japan; ^3^ Graduate School of Medicine Gunma University Maebashi Gunma Japan

## Abstract

Cellular senescence is linked to a wide range of age‐related diseases and can be triggered by a variety of stresses, including DNA damage. A variety of genotoxic stressors, such as anti‐cancer drugs, cause DNA double‐strand breaks (DSBs), which trigger the accumulation of the tumour suppressor protein p53 in the nucleus. Cellular stresses stabilize and activate the p53 signalling pathway, which regulates various cellular processes, such as apoptosis, DNA repair, and senescence. Although p53 signalling is a well‐known tumour suppressor pathway, it remains unclear how it is regulated during cellular senescence. Here, we show that p53‐binding protein 1 (53BP1) accumulation in the nuclear foci is required for DNA damage‐induced cellular senescence via p53 activation. In human immortalized fibroblast, shRNA‐mediated 53BP1 depletion decreased not only the expression of p53‐target genes but also the cellular senescence induced by adriamycin treatment. Furthermore, we confirmed that DSBs trigger the hyperaccumulation of 53BP1 in the nuclear foci, which plays a key role in the regulation of cellular senescence. To prevent the accumulation of 53BP1 in the nuclear foci, we used phase separation inhibitors, and siRNA against RNF168, which accumulates at DSB loci and forms complexes with 53BP1. This blocks the formation of 53BP1 nuclear foci and DNA damage‐induced cellular senescence by activating the p53 signaling pathway. In conclusion,   we demonstrated that increased accumulation of 53BP1 in the nuclear foci following DNA damage activates p53 and governs cellular senescence via a liquid–liquid phase separation mechanism.

## Graphical abstract

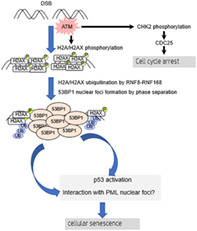

## 1 INTRODUCTION

Cellular senescence is characterized by growth arrest, enlarged nuclei and nucleoli, increased activity of senescence‐associated β‐galactosidase (SA‐β‐gal), formation of senescence‐associated heterochromatic foci, and the induction of senescence‐associated secretory phenotype (SASP) by exogenous and endogenous stimuli, such as telomere shortening, ionizing radiation (IR), and chemotherapeutic agents.^1^, ^2^, ^3^ SASP is thought to be a biologically relevant characteristic of cellular senescence because cytokines, chemokines, growth factors, and extracellular matrix proteases released by senescent cells affect the function of near and distant cells via autocrine/paracrine signalling.^1^, ^2^, ^3^ Cellular senescence is regarded as a barrier against oncogenesis because it is induced by numerous oncogenes, such as mutations in *H‐RAS*, *B‐RAF*, and *c‐MYC* overexpression.^1^, ^2^, ^3^, ^4^, ^5^, ^6^ In this context, oncogenes enhance DNA replication, thereby leading to deregulated DNA replication. For example, unscheduled replication initiation induced by oncogenes causes re‐replication or premature replication origin firing, increasing consumption of the substrates or factors required for the replication (e.g., dNTPs, replication protein A). Consequently, the replication fork is stalled due to a shortage of such replication factors, and tends to collapse.^7^ Deregulated DNA replication also increases replication‐transcription collisions.^8^ Consequently, uncoordinated DNA replication elicits DNA damage, which in turn activates p53, a tumour suppressor gene. p53 induces senescence to prevent the proliferation of cells expressing oncogenes. However, in contrast to the tumour suppressor function of cellular senescence, some reports suggest that it increases oncogenesis, possibly through SASP, via the release of factors that stimulate the proliferation and invasion of tumour cells.^1^, ^2^, ^3^ As another physiological role, cellular senescence is gaining popularity because of its link to health and longevity.^1^, ^2^, ^3^ Senescent cell removal, for example, helps to avoid age‐related disorders and extends healthy life expectancy.^9^, ^10^

The tumour suppressor gene p53 is involved in cellular senescence.^1^, ^2^, ^3^, ^11^ Cellular senescence is caused by a variety of cellular stressors, the most well‐known of which is DNA damage.^1^, ^2^, ^3^ Chemotherapeutic drugs, such as adriamycin (ADR; generic name, doxorubicin), can cause double‐strand breaks (DSBs) in the DNA, which leads to DNA damage‐induced cellular senescence.^12^, ^13^ DSBs activate ataxia telangiectasia mutated (ATM) kinase, which phosphorylates a variety of substrates, including CHK2 kinase. p53 is stabilized and activated by phosphorylation by ATM and/or CHK2, and by post‐translational modification by other factors.^14^, ^15^, ^16^, ^17^ Activated p53 induces the expression of target genes, such as p21, leading to senescence.

53BP1 is a tumour suppressor gene that was discovered as a p53‐binding protein^18^, ^19^ and has received a lot of attention because of its role in DNA damage repair.^20^, ^21^, ^22^, ^23^, ^24^ When DSBs occur, ATM kinase phosphorylates histone H2AX at Ser139 (γH2AX) around the DSB sites, which recruits an adaptor protein, MDC1. MDC1 is also phosphorylated by chromatin‐bound ATM. This MDC1‐phosphorylation recruits the E3 ubiquitin ligase RNF8. L3MBTL2 is also recruited by MDC1 and subsequently poly‐ubiquitinated by RNF8. Afterward, poly‐ubiquitinated L3MBTL2, in turn, facilitates recruitment of the second E3 ubiquitin ligase RNF168 to the DSBs site. RNF168 then catalyses K63‐poly‐ubiquitination of H2A/H2AX. The DSB‐associated poly‐ubiquitin chain of H2A/H2AX recruits 53BP1 and BRCA1, governing the choice between two DSB repair processes, non‐homologous end joining (NHEJ) and homologous recombination (HR), respectively.^20^, ^21^, ^22^, ^25^ 53BP1 is an important positive regulator of NHEJ‐mediated DSB repair during the G1 phase of the cell cycle and protects DSB ends from processing by the DNA end‐resection machinery required for HR‐mediated DSB repair.^20^, ^21^, ^22^, ^25^ In addition to the function related to DSB repair, the p53‐activating function of 53BP1 has been revealed by Cuella‐Martin et al.^26^ When DSB occurs, 53BP1 recruits around DSBs via the mechanism described above and forms nuclear foci (53BP1 nuclear foci). Although the principle mechanism of 53BP1 nuclear foci formation remains unknown, Kilic et al. observed that the liquid–liquid phase separation is essential for 53BP1 nuclear foci formation.^27^ The liquid–liquid phase separation, which has recently emerged as a mechanism to dynamically subdivide the intracellular space, is an important principle for biomolecular condensates and plays a critical role in cell function.^28^ 53BP1 has a variety of activities in response to DNA damage; however, it is unclear whether 53BP1 is involved in DNA damage‐induced senescence or whether 53BP1 nuclear foci formation is required for the phenomenon. In the present study, we demonstrate that DNA damage‐induced senescence requires 53BP1 and 53BP1 nuclear foci formation produced by liquid–liquid phase separation.

## 2 MATERIALS AND METHODS

### 2.1 Cell culture and reagents

The hTERT‐immortalized human fibroblast cell line OUMS‐36T‐3F (obtained from Japanese Collection of Research Bioresources Cell Bank, hereafter referred to as OUMS), the hTERT‐immortalized human retinal pigment epithelial cell line RPE1 (obtained from ATCC), and HEK293T cells (obtained from ATCC) were maintained in Dulbecco's modified Eagle's medium (FUJIFILM Wako Pure Chemical) containing 10% (v/v) fetal bovine serum (Biosera) and penicillin–streptomycin‐glutamine (Thermo Fisher Scientific). Cells were confirmed to be mycoplasma‐free using 4′6‐diamidino‐2‐phenylindole (DAPI) (DAPI Fluoromount‐G, SouthernBiotech). ADR (FUJIFILM Wako Pure Chemical) for DSB‐generation, doxycycline (DOX) (Merck) for inducing shRNA against 53BP1, 1,6‐hexanediol (1,6‐HD) (Sigma‐Aldrich) for inhibition of phase separation, and Sorbitol (Sigma‐Aldrich) for inhibition of phase separation were used at the concentrations indicated in the figure legends.

### 2.2 Plasmids

Tet‐pLKO‐neo (plasmid #21916) was obtained from Addgene. pCAG‐HIVgp and pCMV‐VSV‐G‐RSV‐Rev plasmids were kindly provided by Dr. H. Miyoshi (RIKEN BioResource Center). Tet‐pLKO‐neo‐sh53BP1 was constructed by inserting the annealed oligonucleotides corresponding to the target sequences into AgeI‐EcoRI sites of the Tet‐pLKO‐neo plasmid as described previously.^29^ The target sequence was 5′‐GAACGAGGAGACGGTAATA‐3′.^30^

### 2.3 Virus preparation

Virus preparation was performed as described previously.^29^ Twenty‐four hours before transfection, 1 × 10^6^ HEK293T cells were seeded in a 60‐mm collagen type I‐coated dish (IWAKI, Shizuoka, Japan). Two micrograms of Tet‐pLKO‐neo‐sh53BP1 plasmid were co‐transfected with 1 μg pCAG‐HIVgp and 1 μg pCMV‐VSV‐G‐RSV‐Rev into HEK293T cells using 10 μl Lipofectamine 2000 Transfection Reagent (Thermo Fisher Scientific, Waltham, MA) to produce recombinant lentivirus vector. Seventy‐two hours after transfection, recombinant lentivirus‐containing supernatants were collected.

### 2.4 53BP1‐depletion cells

OUMS/Tet‐on sh53BP1 was obtained as follows. First, OUMS‐36T‐3F cells were infected with 1 ml supernatants containing the recombinant lentiviral vector Tet‐pLKO‐neo‐sh53BP1, for 48 h, and then cells were selected using 1 mg/ml G418 (FUJIFILM Wako Pure Chemical). G418‐resistant cells were seeded in 10‐cm culture dishes (CORNING). A few single cell colonies were picked with a micropipette tip and expanded. One of the expanded cell lines were selected and used in the present study.

### 2.5 Cell survival assay

The cytotoxicity of ADR was assayed using the Cell Counting Kit‐8 according to the manufacturer's protocol (Dojindo).

### 2.6 SA‐β‐gal assay

The SA‐β‐gal assay was performed as previously described.^31^ Briefly, cells were washed with PBS, fixed with 3.7% formaldehyde in PBS for 5 min, and washed twice with PBS. Cells were stained by incubation with SA‐β‐gal staining solution (1 mg/ml 5‐bromo‐4‐chloro‐3‐indolyl‐*β*‐d‐galactoside; 5 mM K_3_Fe(CN)_6_; 5 mM K_4_Fe(CN)_6_; 2 mM MgCl_2_; 150 mM NaCl; 20 mM citric acid; 40 mM Na_2_HPO_4_, pH 6.0) overnight at 37°C. Nuclei were counterstained with KaryoMAX Giemsa staining solution (Invitrogen). Images were obtained using an EVOS™ M5000 imaging system (Thermo Fisher Scientific). More than 300 cells were counted to determine the percentage of SA‐β‐gal‐positive cells. All procedures were performed at room temperature (RT) unless otherwise noted.

### 2.7 Antibodies

Antibodies used in this study are listed in Table S1.

### 2.8 Immunoblotting

Cells were washed with cold PBS at 4°C and directly lysed in Laemmli sample buffer (62.5 mM Tris [pH 6.8]; 2% sodium dodecyl sulfate [SDS]; 20% glycerol). Cell lysates were sonicated with a Branson Sonifier 150 (Branson Ultrasonics) at setting 4 with three 10‐s pulses. After determining the protein concentration of each sample, 2‐mercaptoethanol and bromophenol blue were added to the lysates at concentrations of 5% and 0.025% (v/v), respectively. The lysates were boiled for 7 min and used as whole cell lysates. Equal amounts of protein (3–7 μg) were subjected to electrophoresis using 10% or 4%–20% gradient polyacrylamide gels. The electrophoretically separated proteins were transferred to polyvinylidene difluoride membranes (Pall Corporation) using a submarine transfer apparatus (Criterion Blotter, Bio‐Rad). Immunoblotting was performed using standard procedures.^29^

### 2.9 Immunostaining

Cells cultured in 12‐WELL plates were washed twice with PBS and fixed with 3.7% formaldehyde in PBS for 20 min, followed by permeabilization with 0.3% Triton X‐100 in PBS for 15 min. Cells were then incubated for 30 min in blocking buffer (PBS containing 10% normal goat serum; 3% bovine serum albumin [BSA]; and 0.1% Nonidet P40 [NP40]), followed by incubation in blocking buffer containing the primary antibody (see Table S1 for the descriptions and dilutions of antibodies) overnight. Cells were washed with washing buffer (PBS containing 0.1% NP40) three times and incubated with a secondary antibody conjugated to Alexa 488 and/or Alexa 647 in the dark for 1 h. Images were obtained using an EVOS M5000 Imaging System (Thermo Fisher Scientific) with an EVOS 40X objective lens (fluorite, LWD, 0.65NA/1.79WD). All procedures were performed at RT.

### 2.10 Flow cytometry analysis

The cells were trypsinized and collected in a centrifuge tube. Then, the cells were washed with cold PBS at 4°C once, followed by fixation with 3.7% formaldehyde in PBS for 20 min. After removing formaldehyde, the cells were suspended in PBS containing 0.1% Tween 20 and 1% BSA. BD FACSVCanto II (BD Biosciences) was used to measure the signals of forward scatter (FSC) and side scatter (SSC). Data were analysed using the FlowJo software (Tree Star). All procedures were performed at RT.

### 2.11 RT‐qPCR and data analysis

Total RNA was prepared using the RNeasy Mini kit (Qiagen) and cDNA was synthesized from 0.2–0.5 μg total RNA using a cDNA synthesis kit (FSQ‐101, Toyobo). cDNA was then subjected to RT‐qPCR using pre‐designed gene‐specific primers and probe sets (PrimeTime Std qPCR Assay, IDT) and a reaction mixture (QPS‐101, Toyobo). The primer and probe sequences for the genes are listed in Table S2. Accumulation of PCR products was monitored in real time by measuring the level of fluorescence (PikoReal 96 Real‐Time PCR System, Thermo Fisher Scientific). Results were analysed by the ΔΔC_t_ method and normalized to *GAPDH* using PikoReal software (Thermo Fisher Scientific) to determine relative fold changes in gene expression.

### 2.12 siRNA

The target sequences for siRNAs were as follows: *LacZ*, 5′‐CUCGGCGUUUCAUCUGUGG‐3′ and *RNF168*, 5′‐GAAGAGUCGUGCCUACUGA‐3′.^32^ siRNAs were purchased from Sigma‐Aldrich. siRNAs were transfected with Lipofectamine RNAiMAX (Thermo Fisher Scientific) according to the manufacturer's instructions. Briefly, 5 × 10^4^ cells were seeded on 12‐WELL plates (CORNING), 24 h before transfection. Cells were washed with antibiotics‐free medium and incubated with 1 ml of medium. A volume of 0.75 μl of siRNA (50 μM) and 0.75 μl of RNAiMAX were separately incubated with 125 μl of Opti‐MEM (Thermo Fisher Scientific). After 5 min, the siRNA solution and RNAiMAX solution were mixed and incubated for 20 min. The siRNA/RNAiMAX mixture was added to cells. After 24 h of cell culture (5% CO_2_, 37°C), the mixture was replaced with fresh culture medium.

### 2.13 IdU incorporation assay

Approximately 2 × 10^5^ cells were labelled with 10 μM 5‐Iodo‐2′‐deoxyuridine (IdU, Sigma‐Aldrich) for 1 h. Then, cells were collected in a 1.5‐ml microcentrifuge tube, centrifuged, washed with PBS once, and fixed with 1 ml of 70% ethanol for 24 h at −20°C. After centrifugation, the ethanol was removed and cells were incubated in 200 μl of denature solution (2.5 N HCl; 0.1% Triton X‐100) for 30 min. HCl was removed via centrifugation, and cells were incubated in 400 μl of renature solution (0.1 M Na_2_B_4_O_7_) for 10 min. After centrifugation, cells were incubated in 500 μl of blocking buffer for 30 min, and then incubated with 200 μl of 1/50 diluted anti‐IdU antibody (clone B44, BD biosciences) for 2 h. Cells were washed with washing buffer (1% BSA; 0.1% Tween 100 in BSA) three times and incubated with 200 μl of a secondary antibody solution (1/400 diluted Goat anti‐Mouse IgG [H + L] Cross‐Adsorbed Secondary Antibody, Alexa Fluor™ 488 [Thermo Fisher Scientific]) for 1 h in the dark. Cells were washed with washing buffer three times and incubated with 500 μl of PI solution (10 μM Propidium Iodide [Sigma Aldrich]; 1 mg/ml RNase [NIPPON GENE]) for 1 h. Subsequently, the cells were subjected to flow cytometry analysis. All procedures were performed at RT unless otherwise noted.

### 2.14 Statistical analysis

Student's t‐test and one‐way Analysis of Variance were used for statistical analysis. Each measurement was obtained from more than three independent experiments. Bar plots are represented as means with standard deviations (SD). Statistical significance was set at *p* < 0.05. *p < 0.05; **p < 0.01; ***p < 0.001; ****p < 0.0001. ‘ns’ indicates ‘not significant’.

## 3 RESULTS

### 3.1 53BP1 is required for ADR‐induced cellular senescence

First, we tested whether ADR, a commonly used anti‐cancer treatment, could induce cellular senescence in OUMS cells. A cell survival assay revealed that ADR was very toxic, killing over 80% of cells exposed to above 1000 nM ADR (Figure S1A). In contrast, a senescent condition was generated in OUMS by treating them with 50 nM ADR and chasing them for 7 days (Figure S1B,C). As previously reported, treatment with ADR increased the expression of γH2AX, a DSB marker, in a dose‐dependent manner (Figure S1D).^33^ Based on the above results (Figure S1A–D), we determined that the concentration of ADR to induce cellular senescence in OUMS was 50 nM, the dose used throughout the experiments, or less.

**FIGURE 1:**
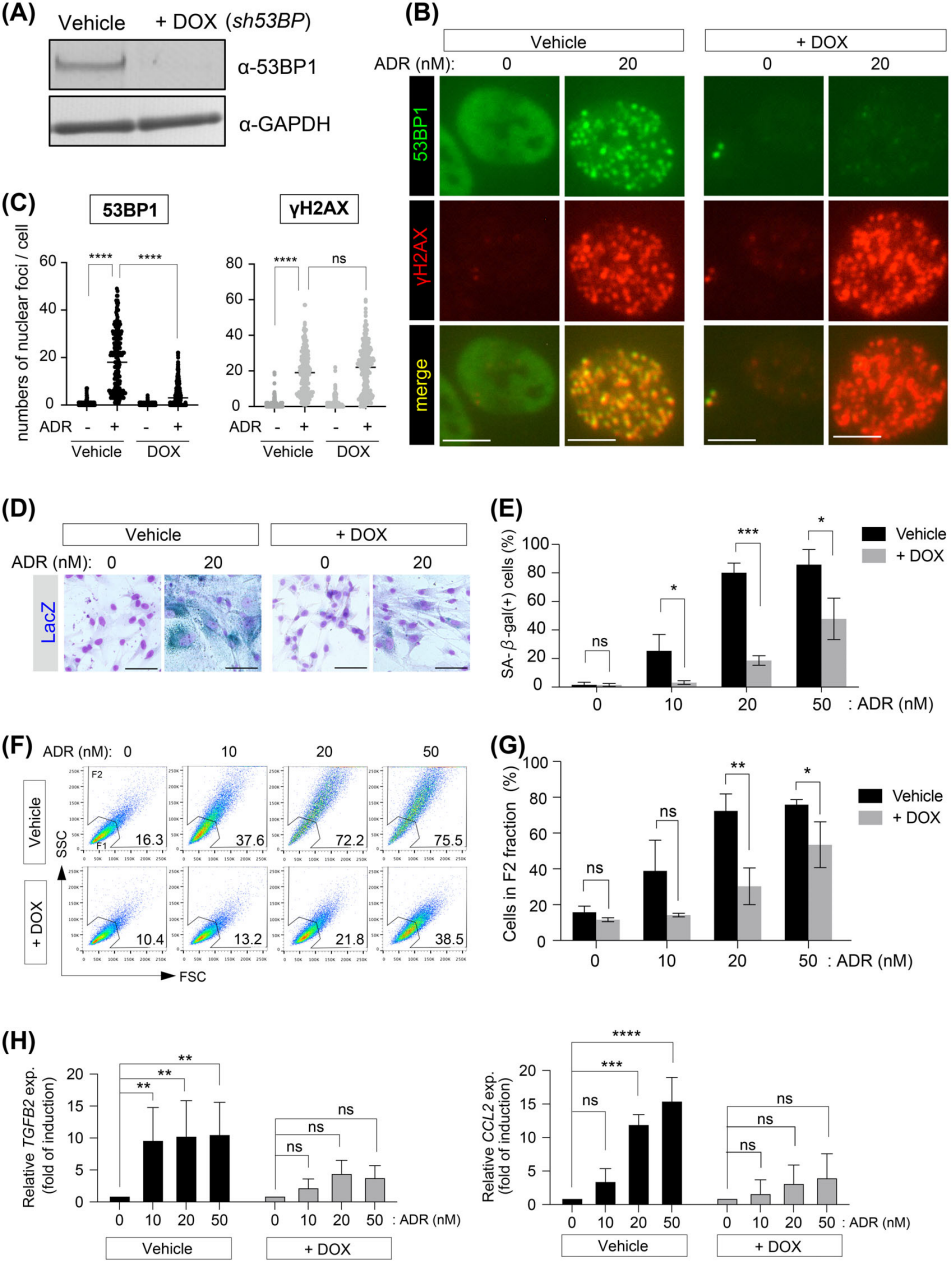
53BP1 depletion suppresses ADR‐induced cellular senescence. (A) OUMS/Tet‐on sh53BP1 cells were incubated with vehicle or DOX for 3 days, and then whole cell lysates were immunoblotted with the indicated antibodies. (B,C) The vehicle‐ or DOX‐incubated cells were treated with 20 nM ADR for 24 h and immunostained with the indicated antibodies. Representative images are shown. Scale bar: 7.5 μm. Dot plot showing the number of 53BP1 and γH2AX nuclear foci per nucleus. Results from three experiments were combined. (D,E) The vehicle‐ or DOX‐incubated cells were treated ADR for 7 days and subjected to the SA‐β‐gal assay. The percentages of SA‐β‐gal‐positive cells are indicated. Values represent the mean ± SD of three independent experiments. Representative images are shown. Scale bar: 60 μm (D). (F,G) Representative flow cytometry results of OUMS cells treated with vehicle or DOX (F). Bar chart of the percentages of senescent cells (F2) with varying ADR treatment. Values represent the mean ± SD of three independent experiments. (H) Relative expression levels of *TGFB2* and *CCL2* determined using RT‐qPCR. Values represent the mean ± SD of three independent experiments. *p < 0.05; **p < 0.01; ***p < 0.001; ****p < 0.0001. ‘ns’ indicates ‘not significant’.

To investigate the role of 53BP1 in DSB‐dependent cellular senescence, we established *53BP1* gene knockdown OUMS cell lines expressing doxycycline (DOX)‐inducible shRNA (OUMS/Tet‐on sh53BP1). DOX treatment reduced 53BP1 levels in cells (Figure 1A). As previously reported, 20 nM ADR caused the development of 53BP1 nuclear foci, which co‐localized with γH2AX nuclear foci in vehicle‐treated (control) cells.^34^ However, when cells were incubated with 20 nM ADR, 53BP1 nuclear foci were not observed, as expected in DOX‐treated cells, despite the presence of γH2AX nuclear foci (Figure 1B,C). We also observed the expression patterns of senescence markers in these cells. The number of SA‐β‐gal‐positive cells increased with ADR concentration in a dose‐dependent manner (Figure 1D,E). On the other hand, the number of SA‐β‐gal‐positive cells was reduced in ADR + DOX‐treated cells (Figure 1D,E). Senescent cells grow in size, and have increased cytoplasmic granularity, which can be detected using flow cytometry that quantifies FSC and SSC.^12^, ^29^, ^35^ Most cells localized in the low FSC and low SSC region at 0 nM ADR, which was defined as fraction 1 (F1) (Figure 1F). In vehicle‐treated cells, ADR increased the number of cells in fraction 2 (F2), which showed higher FSC and SSC compared with that of F1 and contained senescence‐like cells, in a dose dependent manner (Figure 1F,G). The number of ADR‐induced senescent cells in the F2 was reduced when 53BP1 was diminished in the cells by DOX treatment (Figure 1F,G). Furthermore, 53BP1 depletion inhibited the production of SASP factors, such as TGFβ2 and CCL2, as well as other senescence indicators caused by ADR (Figure 1H).

Next, we examined whether 53BP1‐depleted cells continued their growth after ADR‐treatment. The IdU incorporation assay showed that the percentage of IdU‐labelled cells among 53BP1‐depleted cells was lower than that among control cells (43% vs. 30%) under normal conditions, although the underlying mechanism is unclear. (Figure S1E). ADR treatment eliminated IdU‐labelled cells in both control and 53BP1‐depleted cells, suggesting that DNA synthesis was arrested in these cells by the S‐phase checkpoint activated by DSBs (Figure S1E). A DSB‐induced S‐phase checkpoint is typically activated by the ATM‐CHK2‐CDC25A axis,^36^ therefore, we performed an immunoblotting analysis of phosphorylated CHK2 to determine whether this axis is activated. CHK2 was phosphorylated with almost the same intensity in control and 53BP1‐depleted cells (Figure S1F). Taken together, these findings suggest that 53BP1 is required for cellular senescence triggered by ADR, but not required for the ADR‐induced activation of an S‐phase checkpoint under this experimental condition.

### 3.2 53BP1 is involved in ADR‐induced p53 transcriptional activation

**FIGURE 2:**
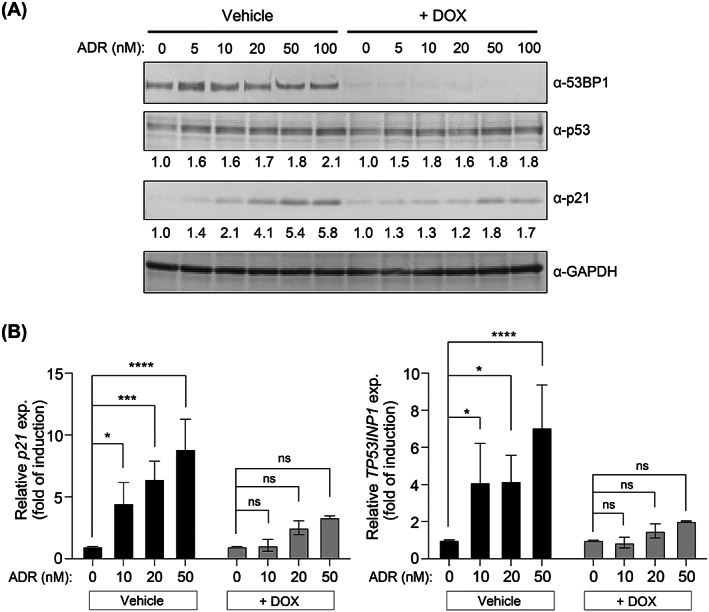
53BP1 depletion suppresses the expression of p53 target genes induced by ADR. (A) Western blot of the relative amounts of p53 and p21 normalized against GAPDH. (B) Expression levels of p53 target genes as determined by RT‐qPCR. Values represent the mean ± SD of three independent experiments. *p < 0.05; ***p < 0.001; ****p < 0.0001. ‘ns’ indicates ‘not significant’.

Since most of the DNA damage‐induced cellular senescence is mediated by p53 activation, we investigated whether 53BP1‐depletion reduces ADR‐induced p53 activation.^1^, ^2^, ^3^, ^11^ ADR increased p21 protein and mRNA levels in a dose‐dependent manner (Figure 2A,B). In contrast, 53BP1‐depletion decreased ADR‐induced p21 expression (Figure 2A,B). Similarly, 53BP1‐depletion decreased the ADR‐induced expression of *TP53INP1*, another p53 target gene (Figure 2B). These findings demonstrate that 53BP1 is necessary for effective ADR‐induced p53 activation.

### 3.3 Phase separation inhibitor blocks both 53BP1 nuclear foci synthesis and ADR‐induced p53 activation

**FIGURE 3:**
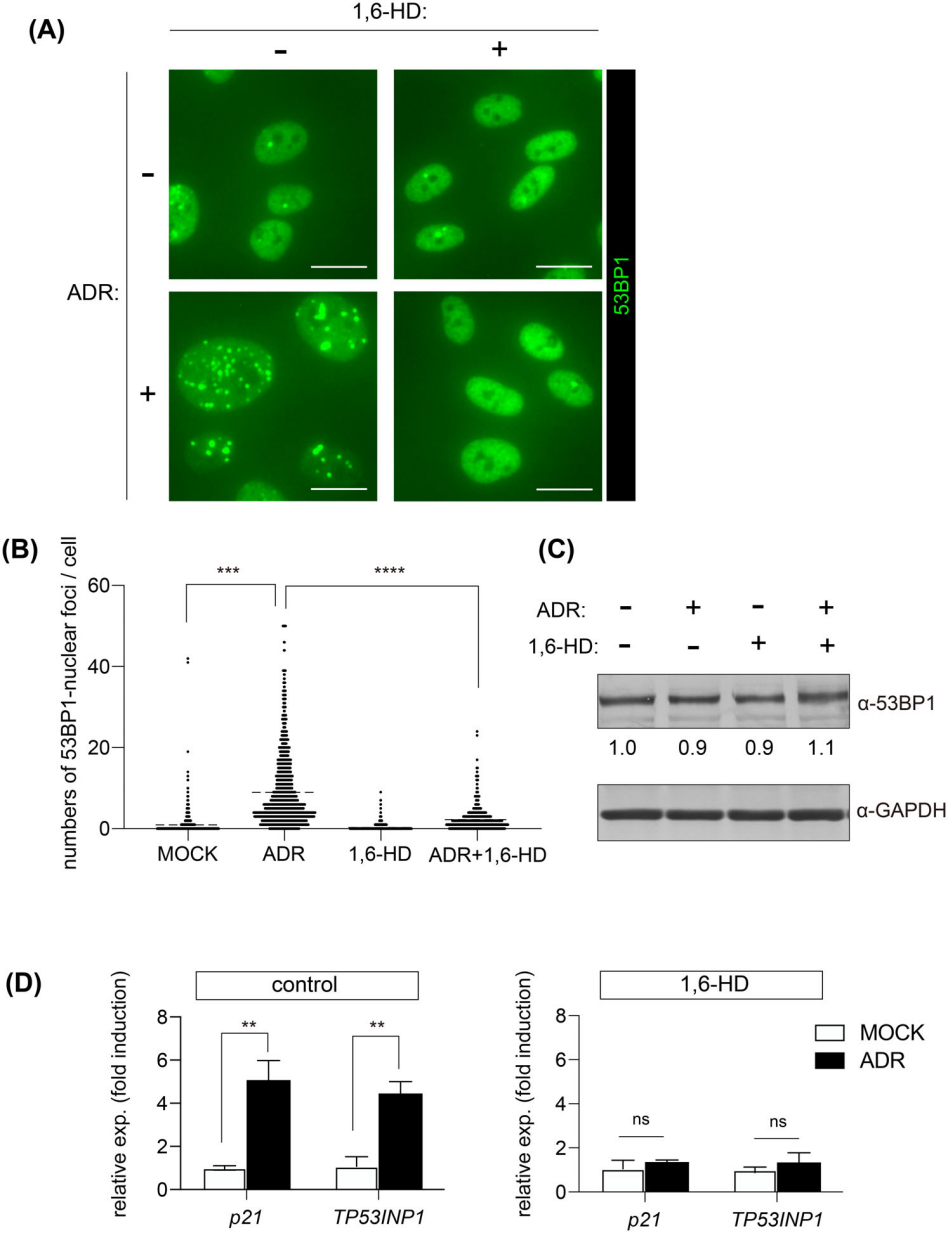
1,6‐hexanediol inhibits ADR‐induced 53BP1 nuclear foci formation and suppresses p53 activation. (A,B) OUMS/Tet‐on sh53BP1 cells were incubated with 0.3% 1,6‐hexanediol and/or 20 nM ADR for 24 h. (A) Representative images of cells immunostained with anti‐53BP1 antibody. Scale bar: 15 μm. (B) Dot plot showing the number of 53BP1 nuclear foci per nucleus. Results from three experiments were combined. (C) Whole cell lysates from the cells treated with 1,6‐hexanediol and/or ADR immunoblotted with anti‐53BP1 antibodies. The relative amounts of 53BP1 normalized against GAPDH are shown. (D) Bar chart of ADR‐induced fold‐changes in p53 target genes in the absence or presence of 1,6‐hexanediol. Values represent the mean ± SD of three independent experiments. **p < 0.01; ***p < 0.001; ****p < 0.0001. ‘ns’ indicates ‘not significant’.

IR‐induced p53 activation requires 53BP1 nuclear foci, according to a recent study.^27^ We examined whether 53BP1 nuclear foci is also necessary for ADR‐induced p53 activation. Liquid–liquid phase separation, which has recently emerged as a mechanism to dynamically subdivide the intracellular space, produces 53BP1 nuclear foci.^27^, ^28^ Because 1,6‐hexanediol (1,6‐HD) has been shown to suppress the formation of 53BP1 nuclear foci induced by IR,^27^ we used 1,6‐HD and/or ADR to treat cells. The number of 53BP1 nuclear foci in ADR‐administrated cells increased compared to control cells (Figure 3A,B). In contrast, 1,6‐HD significantly reduced the number of 53BP1 nuclear foci (Figure 3A,B). The addition of 1,6‐HD had no effect on the expression level of 53BP1 protein under these conditions (Figure 3C).

Next, we examined the effect of 1,6‐HD on ADR‐induced p53 activation. In ADR‐treated cells, the expression of *p21* and *TP53INP1* increased by approximately 5.1 and 4.5 times, respectively (Figure 3D). However, in the presence of 1,6‐HD, ADR‐induced expression of these genes was virtually repressed (Figure 3D). Similar results were obtained using immortalized human retinal pigment epithelial cells, RPE1 (Figure S2A–C). These results suggest that ADR‐induced p53 activation requires the formation of 53BP1 nuclear foci.

### 3.4 ADR‐induced senescence is suppressed by a phase separation inhibitor

**FIGURE 4:**
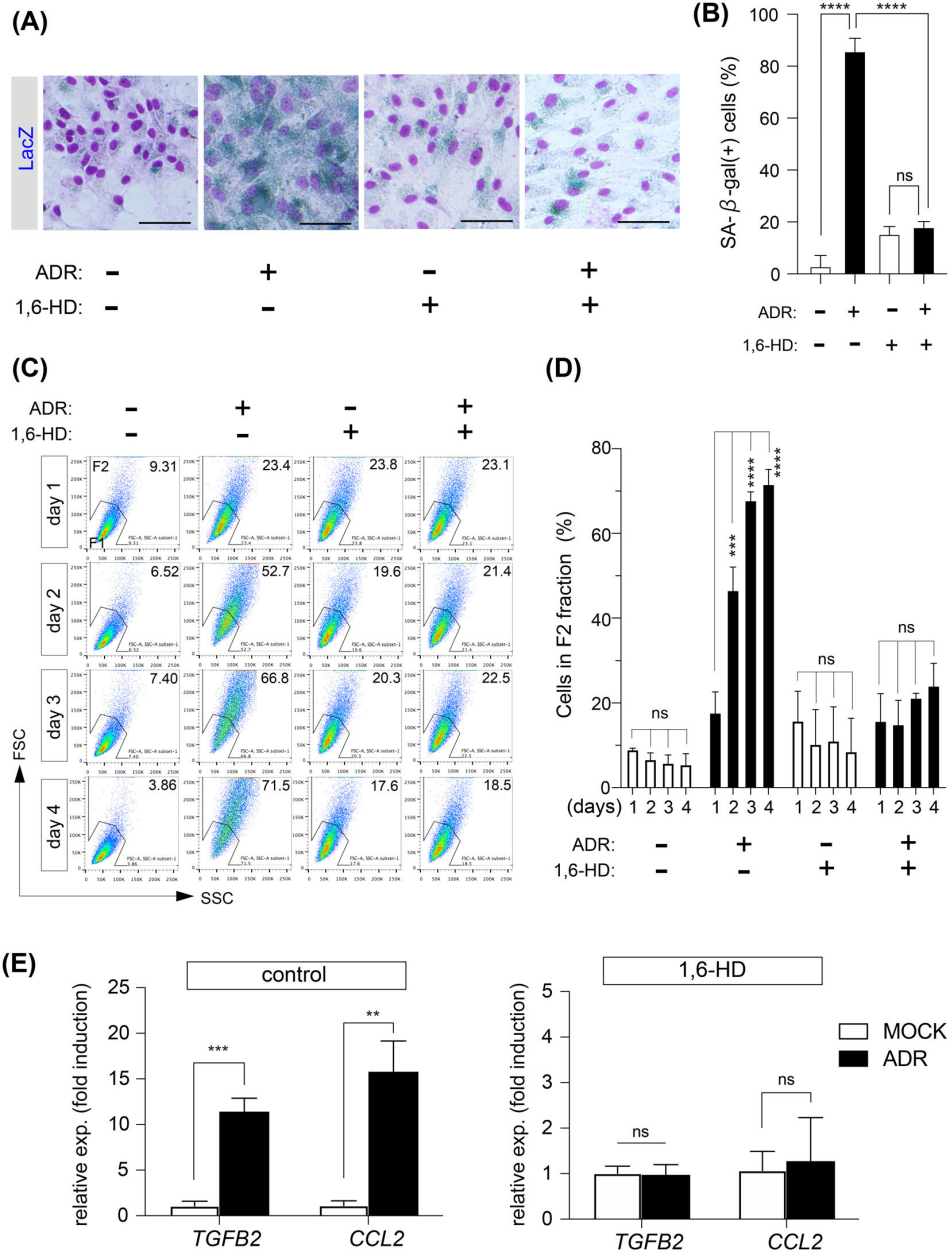
1,6‐hexanediol suppresses ADR‐induced cellular senescence. (A,B) Representative images from the SA‐β‐gal assay of OUMS/Tet‐on sh53BP1 cells treated with 0.3% 1,6‐hexanediol and/or 20 nM ADR for 4 days. Scale bar: 60 μm (A). Percentages of SA‐β‐gal‐positive cells. Values represent the mean ± SD of three independent experiments. (C,D) Representative results of flow cytometry analyses of OUMS/Tet‐on sh53BP1 cells (C). The percentage of senescent cells (F2) among OUMS/Tet‐on sh53BP1 cells. Values represent the mean ± SD of three independent experiments. (E) Bar chart of ADR‐induced fold‐changes in *TGFB2* and *CCL2* in the absence or presence of 1,6‐hexanediol. Values represent the mean ± SD of three independent experiments. **p < 0.01; ***p < 0.001; ****p < 0.0001. ‘ns’ indicates ‘not significant’.

We investigated whether 1,6‐HD suppresses ADR‐induced senescence by inhibiting the 53BP1 nuclear foci formation and p53 activation. ADR enhanced the number of SA‐β‐gal‐positive cells by approximately 86% (Figure 4A,B). However, 1,6‐HD reduced the number of ADR‐induced SA‐β‐gal‐positive cells to approximately 18% (Figure 4A,B). Flow cytometry analysis revealed that ADR increased the number of morphologically senescent cells in a time‐dependent manner (Figure 4C,D). On Day 4, 1,6‐HD decreased the number of morphologically senescent cells induced by ADR from about 71% to about 24% (Figure 4D). In comparison to only ADR‐treated cells, administration of 1,6‐HD generated morphologically senescent cells up to approximately 16% on Day 1; however, the percentage of morphologically senescent cells did not increase until Day 4 (Figure 4D). The morphological alteration caused by 1,6‐HD could be related to the disturbance of cytoskeletal organization.^37^ In addition to the senescence indicators described above, 1,6‐HD inhibited the expression of ADR‐induced SASP factors (Figure 4E). Taken together, these results suggest that 1,6‐HD prevents ADR‐induced cellular senescence by affecting the formation of 53BP1 nuclear foci, which suppresses p53 activation. The effects of 1,6‐HD on ADR‐induced senescence were also observed in RPE1 cells, except *CCL2* mRNA expression. CCL2 may not be an SASP factor in RPE1 cells (Figure S3A,B,C,D,E). In addition to 1,6‐HD, Sorbitol, a known phase separation inhibitor,^27^ prevented 53BP1 nuclear foci formation, p53 activation, and cellular senescence in OUMS/Tet‐on sh53BP1 cells (Figure S4A,B,C,D,E).

### 3.5 Depletion of RNF168 inhibited ADR‐induced 53BP1 nuclear foci formation and p53 activation

**FIGURE 5:**
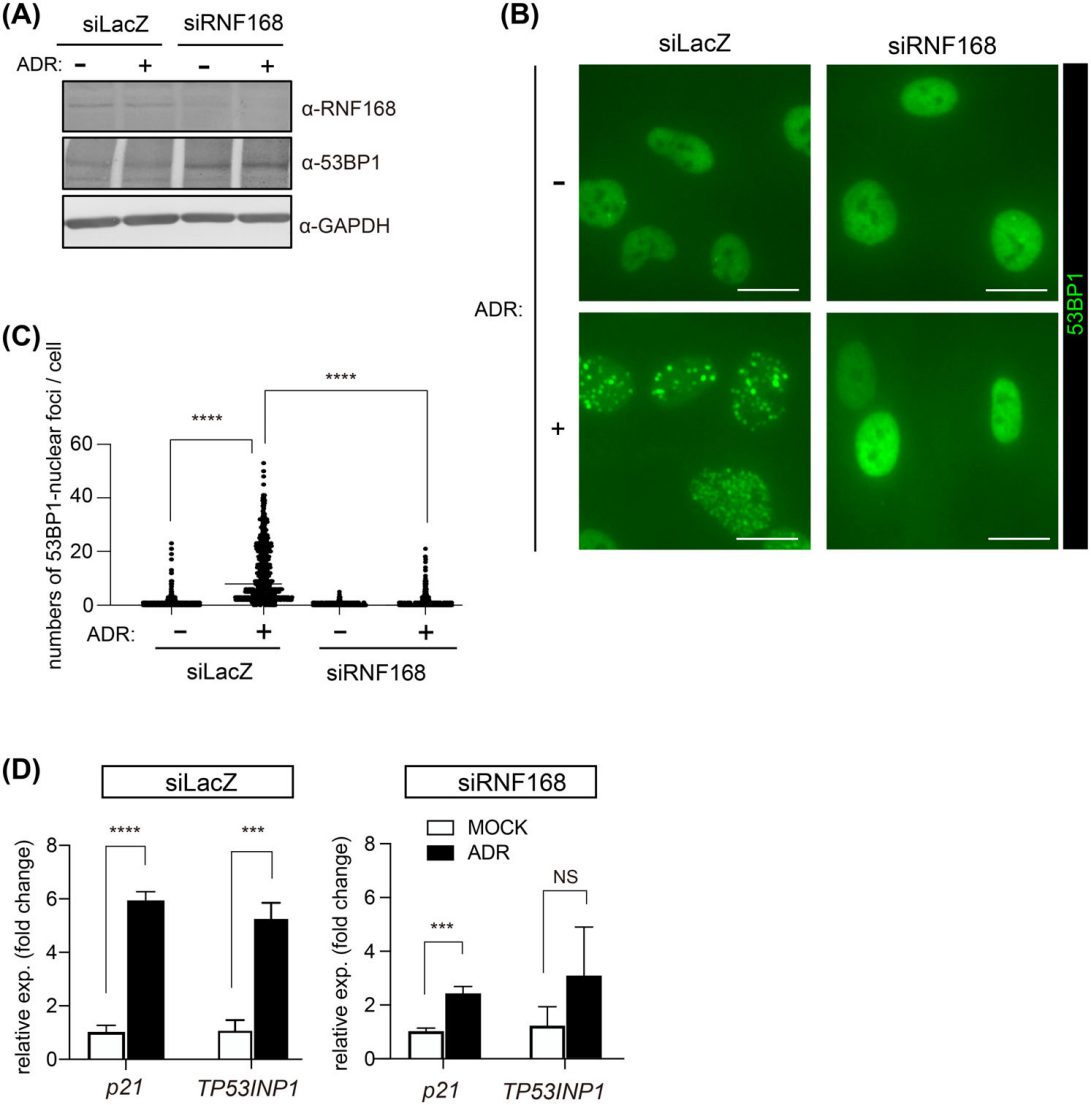
RNF168 depletion inhibits ADR‐induced 53BP1 nuclear foci formation and suppresses p53 activation. OUMS/Tet‐on sh53BP1 cells transfected with siLacZ or siRNF168 were cultured for 2 days, and then cells were treated with vehicle (−) or 20 nM ADR (+) for 24 h. (A) Western blot of OUMS/Tet‐on sh53BP1 whole cell lysates immunoblotted with anti‐RNA168 and anti‐53BP1 antibodies. (B,C) Representative images of cells immunostained with anti‐53BP1 antibody. Scale bar: 15 μm (B). Dot plot showing the number of 53BP1 nuclear foci per nucleus. Results of three experiments were combined. (D) Bar chart showing ADR‐induced fold‐changes in p53 targets genes in the presence or absence of RNF168. Values represent the mean ± SD of three independent experiments. ***p < 0.001; ****p < 0.0001. ‘ns’ indicates ‘not significant’.

To further confirm that 53BP1 nuclear foci formation is required for ADR‐induced senescence via p53 activation, we employed RNF168‐depleted cells (Figures 5A and S5A). RNF168 is an E3 ligase that has been linked to the formation of 53BP1 nuclear foci in response to DNA damage.^32^, ^38^ RNF168 siRNA depletion did not reduce 53BP1 levels (Figure 5A), but it did impair ADR‐induced 53BP1 nuclear foci formation, as reported previously (Figure 5B,C).^32^, ^38^ ADR‐induced p53 activation was controlled, which is compatible with the formation of 53BP1 nuclear foci. In siLacZ‐transfected cells, ADR enhanced the expression of *p21* and *TP53INP1* (Figure 5D). In contrast, the ADR‐induced expression of p53 target genes was decreased in *RNF168*‐depleted cells (Figure 5D). Similar results were obtained using RPE1 cells (Figure S5A–D). These findings, in addition to those obtained with 1,6‐HD, suggest that 53BP1 nuclear foci formation is required for ADR‐induced p53 activation.

### 3.6 ADR‐induced cellular senescence decreased with RNF168 depletion

**FIGURE 6:**
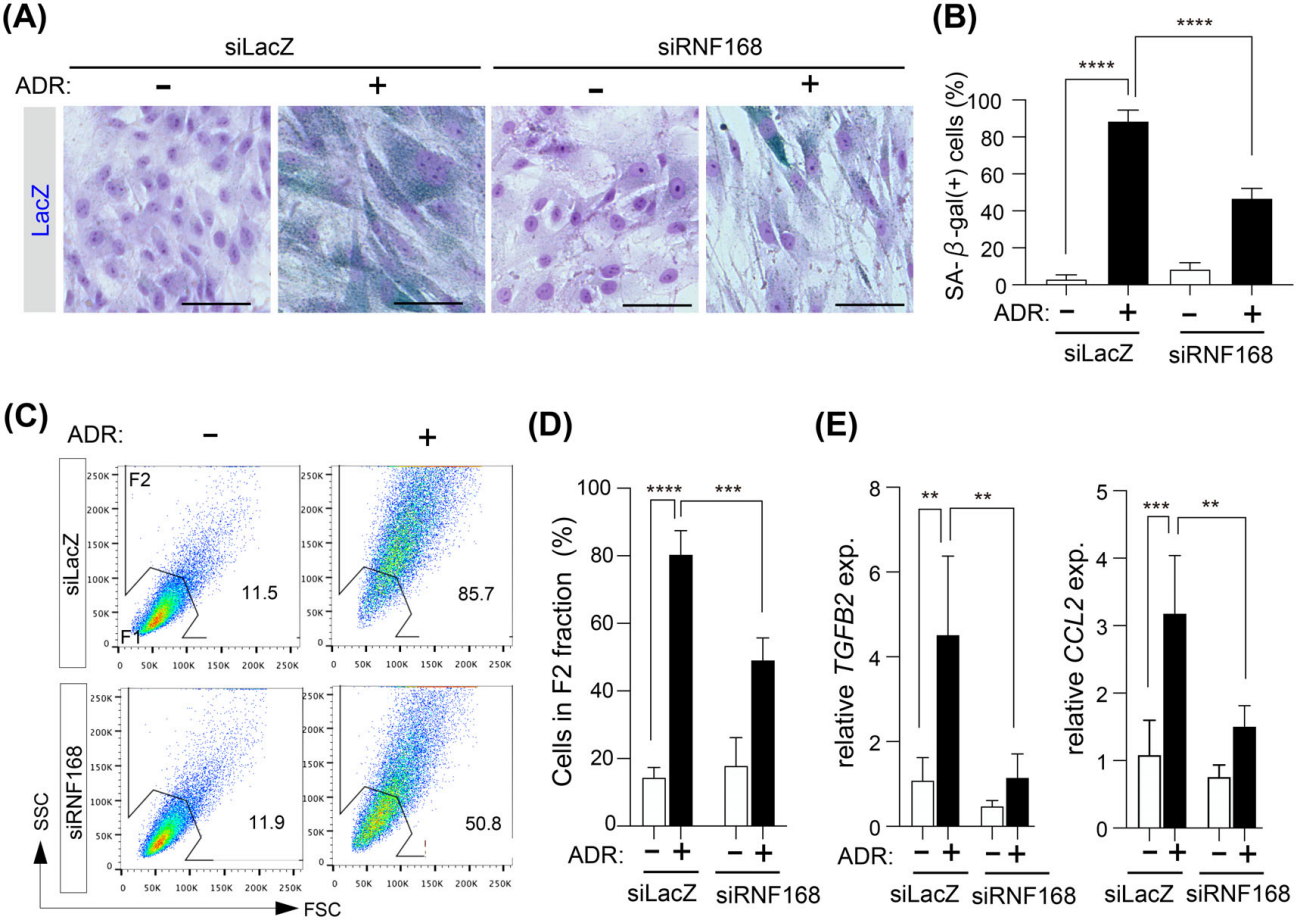
RNF168 depletion suppresses ADR‐induced cellular senescence. (A,B) OUMS/Tet‐on sh53BP1 cells transfected with siLacZ or siRNF168 were cultured for 24 h, and then cells were treated with vehicle (−) or 20 nM ADR (+) for 6 days. Representative images of SA‐β‐gal staining. Scale bar: 60 μm (A). Percentages of SA‐β‐gal‐positive cells. Values represent the mean ± SD of three independent experiments. (C,D) OUMS/Tet‐on sh53BP1 cells were transfected with siRNA. After 2 days, a second siRNA transfection was performed. Six hours after the second transfection, cells were incubated with vehicle (−) or 20 nM ADR (+) for 3 days. Representative results of flow cytometry analyses (C). Percentages of senescent cells (F2). Values represent the mean ± SD of three independent experiments. (E) Bar chart showing ADR‐induced fold‐changes in *TGFB2* and *CCL2* in the presence or absence of RNF168. Values represent the mean ± SD of three independent experiments. **p < 0.01; ***p < 0.001; ****p < 0.0001.

Finally, we examined whether ADR‐induced cellular senescence was suppressed in RNF168‐depleted cells that did not produce 53BP1 nuclear foci. ADR increased the number of SA‐β‐gal‐positive cells in siLacZ‐transfected cells up to 89%, and it only increased the number of SA‐β‐gal‐positive cells in RNF168‐depleted cells by approximately 47% (Figure 6A,B). Similarly, whereas ADR enhanced the morphological changes associated with senescence in siLacZ‐transfected cells to around 80%, it only produced approximately 49% the morphologically senescent cells in RNF168‐depleted cells (Figure 6C,D). ADR‐induced SASP factor expression was decreased in RNF168‐depleted cells relative to siLacZ‐transfected cells, which was consistent with SA‐β‐gal expression and morphological alterations (Figure 6E). Similar results were obtained using RPE1 cells (Figure S6A–E). These results further suggest that the p53‐mediated cellular senescence induced by ADR requires the formation of 53BP1 nuclear foci.

## 4 DISCUSSION

Although it was first identified as a p53‐binding protein, 53BP1 has been studied as a DNA damage repair protein that enhances the NHEJ‐mediated DSB repair pathway.^20^, ^21^, ^22^, ^23^, ^39^ Recently, 53BP1 has been shown to activate p53 independently of its DNA repair function.^26^ In this study, we examined the involvement of 53BP1 in DNA damage‐induced cellular senescence. We showed that 53BP1 protein levels and the formation of 53BP1 nuclear foci are critical for DNA damage‐induced cellular senescence via p53 activation.

ADR increased the number of 53BP1 nuclear foci co‐localized with γH2AX nuclear foci, and 53BP1‐depletion had little effect on the formation of γH2AX nuclear foci. Therefore, 53BP1 nuclear foci formation occurred after the formation of γH2AX nuclear foci, as previously reported.^27^ In addition to the disappearance of 53BP1 nuclear foci, 53BP1‐depletion suppressed the expression of senescence markers, such as SA‐β‐gal, morphological changes, and SASP factors. In this study, we determined that the expression of p53‐target genes including *p21* and *TP53INP1* was also suppressed, suggesting that 53BP1 is required for the activation of p53, as previously reported.^26^, ^27^ Therefore, 53BP1 is required for DNA damage‐induced cellular senescence mediated by p53 activation. Since 53BP1‐depletion decreased not only its protein levels, but also 53BP1 nuclear foci, it was difficult to evaluate the effect of 53BP1 nuclear foci formation on ADR‐induced cellular senescence. Therefore, we disrupted 53BP1 nuclear foci formation using the phase separation inhibitor 1,6‐HD. 1,6‐HD is an aliphatic alcohol and has been reported to inhibit IR‐induced 53BP1 nuclear foci formation.^27^ 1,6‐HD suppressed ADR‐induced expression of p53‐target genes. In addition, it suppressed the expression of senescence markers induced by ADR. Therefore, 53BP1 nuclear foci formation is required for ADR‐induced p53 activation and subsequent induction of cellular senescence. The E3 ligase RNF168 is recruited to DSB sites and is required for 53BP1 nuclear foci formation.^32^, ^38^ We inhibited 53BP1 nuclear foci formation by depleting RNF168 and confirmed that 53BP1 nuclear foci formation was required for ADR‐induced senescence. In addition to RNF168, several factors have been reported to regulate 53BP1 nuclear foci formation,^40^, ^41^, ^42^, ^43^, ^44^, ^45^ which might also regulate DNA damage‐induced senescence.

We cannot exclude the possibility that 53BP1 nuclear foci may have another function in ADR‐induced cellular senescence, aside from its role in p53 activation. 53BP1 nuclear foci could possibly interact with other nuclear structures. Promyelocytic leukaemia protein (PML) is reported to be involved in cellular senescence.^46^, ^47^ Overexpression of PML induces cellular senescence and forms a subnuclear structure called PML nuclear foci.^48^ In addition, PML nuclear foci are formed in response to DSB elicited by RAS or IR.^48^, ^49^ PML nuclear foci co‐localizes with 53BP1 nuclear foci in the nucleus, in DSB sites marked by γH2AX.^49^, ^50^, ^51^ 53BP1 nuclear foci activates p53 and may interact with PML nuclear foci to induce optimized cellular senescence (Figure S7).

Intranuclear structures formed by liquid–liquid phase separation have attracted much attention in recent years.^28^ To the best of our knowledge, this is the first time that liquid–liquid phase separation was shown to be involved in cellular senescence. We previously reported a mechanism of p53 dependent cellular senescence induced by HSF1‐depletion.^29^ In HSF1‐depletion‐induced cellular senescence, the time course of p53 activation and expression of senescence markers is slower than that of DNA damage‐induced senescence. Notably, the increased 53BP1 nuclear foci formation observed in ADR‐treated cells was not observed in HSF1‐depleted cells (data not shown). Therefore, the rapid and abundant formation of 53BP1 nuclear foci in response to DSBs may have a role in the fast induction of cellular senescence.

In the present study, we used two immortalized normal human cell lines derived from fibroblasts and retinal pigment epithelial cells to show that 53BP1 nuclear foci formation is required for DNA damage‐induced senescence. Although we could not detect marked differences in numbers and sizes of 53BP1 nuclear foci, or foci formation kinetics in response to ADR between the two cell lines under our experimental conditions (data not shown), determining whether such parameters of 53BP1 foci influence the DNA damage‐induced senescence would be insightful. For example, recently, Bobkova et al. reported that 53BP1 nuclear foci are formed at different levels of efficiency in normal human skin fibroblasts and U87 glioblastoma cell lines,^52^ and both have wild‐type p53. In U87 cells, 53BP1 nuclear foci induced by IR appeared less compact and more dispersed than those in normal human skin fibroblasts. Whether such differences are related to the efficiency of induction of cellular senescence will be explored in future studies. Cellular response to DNA‐damaging agents is dependent on the amount of DSBs. A high dose of ADR exerted cytotoxicity (Figure S1A), which could be due to unrepairable DSBs. In contrast, we demonstrated that a low dose of ADR induced cellular senescence. These cellular responses were also observed in cells treated with other DNA‐damaging agents, such as camptothecin, etoposide, and gemcitabine (data not shown). After removal of the low concentrations of DNA‐damaging agents, some cells, in which DSBs might have been repaired, began to proliferate again (data not shown). In addition, a low‐dose of ADR activated the ATM‐CHK2‐CDC25A axis and p53, and induced S‐phase arrest and senescence, respectively. Our data indicate that 53BP1 is required for cellular senescence, but not for S‐phase arrest (Figure S7).

## AUTHOR CONTRIBUTIONS

Tsukasa Oda and Nobuo Sasaki designed research; Tsukasa Oda and Nanami Gotoh performed research and analysed data; Tsukasa Oda and Nobuo Sasaki wrote the article; Tetsuhiro Kasamatsu, Hiroshi Handa, and Takayuki Saitoh contributed new reagents or analytic tools. All authors have read and approved the final manuscript.

## FUNDING INFORMATION

This work was supported by the Japan Society for Promotion Science (JSPS) KAKENHI (Grant number JP15K06866 to Tsukasa Oda, and JP19H03455, JP19K22544 to Nobuo Sasaki), and by Japan Agency for Medical Research and Development (AMED) (Grant number JP21ae0121046, to Nobuo Sasaki). Financial support was provided by the Takeda Science Foundation (Tsukasa Oda).

## CONFLICT OF INTEREST

The authors declare no competing or financial interest.

## Supporting information


**Table S1.** List of antibodies.

**Table S2.** Sequences of primers and probes used for the qPCR.

**Figure S1.** Low‐dose of ADR induces cell‐cycle arrest and cellular senescence. (A) Cell survival assay of OUMS/Tet‐on sh53BP1 cells treated with the indicated concentrations of ADR for 3 days. Values represent the mean ± of SD of triplicate wells. Similar results were obtained from two independent experiments. (B) Representative images of SA‐β‐gal assay using OUMS/Tet‐on sh53BP1 cells treated with the indicated concentrations of ADR for 7 days Scale bar: 60 μm. (C) Percentages of SA‐β‐gal‐positive cells. (D) Western blot of whole cell lysates of OUMS/Tet‐on sh53BP1 cells treated with the indicated concentrations of ADR for 24 h. The relative amounts of γH2AX normalized against H2AX are shown. (E) Representative results of flow cytometry analyses of IdU‐labelled OUMS/Tet‐on sh53BP1 cells. The rectangle represents IdU‐labelled cells and its percentages. Similar results were obtained from two independent experiments. (F) OUMS/Tet‐on sh53BP1 cells were incubated with vehicle or DOX for 3 days, and then treated with ADR for 24 h. Whole cell lysates were immunoblotted with the indicated antibodies.
**Figure S2.** 1,6‐hexanediol inhibits ADR‐induced 53BP1 nuclear foci formation and suppresses p53 activation in RPE1 cells. (A,B) RPE1 cells were incubated with 0.3% 1,6‐hexanediol and/or 20 nM ADR for 24 h. Representative images of cells immunostained with an anti‐53BP1 antibody. Scale bar: 15 μm (A). Dot plot showing the number of 53BP1 nuclear foci per nucleus. Results from three experiments were combined. (C) Bar chart showing ADR‐induced fold‐changes in p53 target genes in the absence or presence of 1,6‐hexanediol. Values represent the mean ± SD of three independent experiments. **p < 0.01; ****p < 0.0001. ‘ns’ indicates ‘not significant’.
**Figure S3.** 1,6‐hexanediol suppresses ADR‐induced cellular senescence in RPE1 cells. (A,B) Representative images from the SA‐β‐gal assay of RPE1 cells treated with 0.3% 1,6‐hexanediol and/or 20 nM ADR for 5 days. Scale bar: 60 μm (A). Percentages of SA‐β‐gal‐positive cells. Values represent the mean ± SD of three independent experiments. (C,D) Representative results of flow cytometry analyses of RPE1 cells. (C) F2 indicates morphologically senescent cells among RPE1 cells. Values represent the mean ± SD of three independent experiments. (E) Bar chart showing ADR‐induced fold‐changes in *TGFB2* and *CCL2* in the absence or presence of 1,6‐hexanediol. Values represent the mean ± SD of three independent experiments. **p < 0.01; ***p < 0.001; ****p < 0.0001. ‘ns’ indicates ‘not significant’.
**Figure S4.** Sorbitol suppresses 53BP1 nuclear foci formation, p53 activation, and SA‐β‐gal expression induced by ADR. (A,B) OUMS/Tet‐on sh53BP1 cells were incubated with 0.3 M Sorbitol and/or 20 nM ADR for 24 h. Representative images of cells immunostained with anti‐53BP1 antibody. Scale bar: 15 μm (A). Dot plot showing the number of 53BP1 nuclear foci per nucleus. (C) Bar chart showing ADR‐induced fold‐changes in p53 target genes in the absence or presence of Sorbitol. (D,E) OUMS/Tet‐on sh53BP1 cells treated with 0.3 M Sorbitol and/or 20 nM ADR for 4 days. (D,E) Representative images from the SA‐β‐gal assay. Scale bar: 60 μm (D). Percentages of SA‐β‐gal‐positive cells. ****p < 0.0001.
**Figure S5.** RNF168 depletion inhibits ADR‐induced 53BP1 nuclear foci formation and suppresses p53 activation. (A) OUMS/Tet‐on sh53BP1 cells or RPE1 cells were transfected with siRNA against LacZ or RNF168. After 48 h, relative expression levels of RNF168 mRNA were examined. (B,C) Representative images of cells immunostained with an anti‐53BP1 antibody. Scale bar: 15 μm (B). Dot plot showing the number of 53BP1 nuclear foci per nucleus. Results of three experiments were combined. (D) Bar chart showing ADR‐induced fold‐changes in p53 targets genes in the presence or absence of RNF168. Values represent the mean ± SD of three independent experiments. *p < 0.05; **p < 0.01; ***p < 0.001; ****p < 0.0001. ‘ns’ indicates ‘not significant’.
**Figure S6.** RNF168 depletion suppresses ADR‐induced cellular senescence in RPE1 cells. (A,B) RPE1 cells transfected with siLacZ or siRNF168 were cultured for 24 h, and the cells were then treated with vehicle (−) or 20 nM ADR (+) for 5 days. Representative images of SA‐β‐gal staining. Scale bar: 60 μm (A). Percentages of SA‐β‐gal‐positive cells. Values represent the mean ± SD of three independent experiments. (C,D) RPE1 cells were transfected with siRNA. After 24 h, cells were incubated with vehicle (−) or 20 nM ADR (+) for 5 days. Representative results of flow cytometry analyses. (C) Percentages of senescent cells (F2). Values represent the mean ± SD of three independent experiments. (E) Bar chart showing ADR‐induced fold‐changes in *TGFB2* and *CCL2* in the presence or absence of RNF168. Values represent the mean ± SD of three independent experiments. **p < 0.01; ***p < 0.001; ****p < 0.0001. ‘ns’ indicates ‘not significant’.
**Figure S7.** Role of 53BP1 nuclear foci in DSB‐induced cellular senescence. ATM activated by DSBs phosphorylates CHK2 and H2A/H2AX. ATM and CHK2 induce S‐phase cell cycle arrest via CDC25. Phosphorylated H2A/H2AX around the DSB sites are subsequently ubiquitinated by RNF‐8‐RNF168. Then, 53BP1 is accumulated at the DSB sites, and 53BP1 nuclear foci is formed by phase separation. 53BP1 nuclear foci participates DSB‐induced cellular senescence via p53 activation. 53BP1 nuclear foci may also interact with other nuclear factors, such as PML nuclear foci, to regulate cellular senescence.

## Data Availability

The data that support the findings of this study are available from the corresponding author upon reasonable request.
