# Supplementary material for: DNA damage‐induced cellular senescence is regulated by 53BP1 accumulation in the nuclear foci and phase separation
Source: Cell Prolif. 2023 Jan 15;56(6):e13398. doi: 10.1111/cpr.13398 (PMC10280147; doi:10.1111/cpr.13398)
Supplement: Supplementary file 1 — Table S1. List of antibodies. [file CPR-56-e13398-s001.docx]

**Table S1 List of antibodies.**

| **Antibody** | **Catalog Number** | **Dilution** |
| --- | --- | --- |
| Rabbit polyclonal anti-53BP1 | Calbiochem, PC712 | 1:5000 (WB), 1:9000 (IF) |
| Rabbit monoclonal anti-GAPDH | Cell Signaling Technology, 2118 | 1:10000 (WB) |
| Mouse monoclonal anti-γH2AX | Merck, 05-636 | 1:1000 (WB), 1:800 (IF) |
| Mouse monoclonal anti-p53 | Santa Cruz Biotechnology, sc-126 | 1:1000 (WB) |
| Rabbit monoclonal anti-p21 | Cell Signaling Technology, 2947 | 1:1000 (WB) |
| Mouse monoclonal anti-RNF168 | Santa Cruz Biotechnology, sc-101125 | 1:1000 (WB) |
| Rabbit monoclonal anti-H2AX | Cell Signaling Technology, 7631 | 1:1000 (WB) |
| Rabbit monoclonal anti-CHK2 | Cell Signaling Technology, 6334 | 1:1000 (WB) |
| Rabbit polyclonal anti-pCHK2 (T68) | Cell Signaling Technology, 2661 | 1:1000 (WB) |
