# Supplementary material for: DNA damage‐induced cellular senescence is regulated by 53BP1 accumulation in the nuclear foci and phase separation
Source: Cell Prolif. 2023 Jan 15;56(6):e13398. doi: 10.1111/cpr.13398 (PMC10280147; doi:10.1111/cpr.13398)
Supplement: Supplementary file 3 — Figure S1. Low‐dose of ADR induces cell‐cycle arrest and cellular senescence. (A) Cell survival assay of OUMS/Tet‐on sh53BP1 cells treated with the indicated concentrations of ADR for 3 days. Values represent the mean ± of SD of triplicate wells. Similar results were obtained from two independent experiments. (B) Representative images of SA‐β‐gal assay using OUMS/Tet‐on sh53BP1 cells treated with the indicated concentrations of ADR for 7 days Scale bar: 60 μm. (C) Percentages of SA‐β‐gal‐positive cells. (D) Western blot of whole cell lysates of OUMS/Tet‐on sh53BP1 cells treated with the indicated concentrations of ADR for 24 h. The relative amounts of γH2AX normalized against H2AX are shown. (E) Representative results of flow cytometry analyses of IdU‐labelled OUMS/Tet‐on sh53BP1 cells. The rectangle represents IdU‐labelled cells and its percentages. Similar results were obtained from two independent experiments. (F) OUMS/Tet‐on sh53BP1 cells were incubated with vehicle or DOX for 3 days, and then treated with ADR for 24 h. Whole cell lysates were immunoblotted with the indicated antibodies. Figure S2. 1,6‐hexanediol inhibits ADR‐induced 53BP1 nuclear foci formation and suppresses p53 activation in RPE1 cells. (A,B) RPE1 cells were incubated with 0.3% 1,6‐hexanediol and/or 20 nM ADR for 24 h. Representative images of cells immunostained with an anti‐53BP1 antibody. Scale bar: 15 μm (A). Dot plot showing the number of 53BP1 nuclear foci per nucleus. Results from three experiments were combined. (C) Bar chart showing ADR‐induced fold‐changes in p53 target genes in the absence or presence of 1,6‐hexanediol. Values represent the mean ± SD of three independent experiments. **p < 0.01; ****p < 0.0001. ‘ns’ indicates ‘not significant’. Figure S3. 1,6‐hexanediol suppresses ADR‐induced cellular senescence in RPE1 cells. (A,B) Representative images from the SA‐β‐gal assay of RPE1 cells treated with 0.3% 1,6‐hexanediol and/or 20 nM ADR for 5 days. Scale bar: 60 μm (A). Percent [file CPR-56-e13398-s002.pptx]

## Slide 1
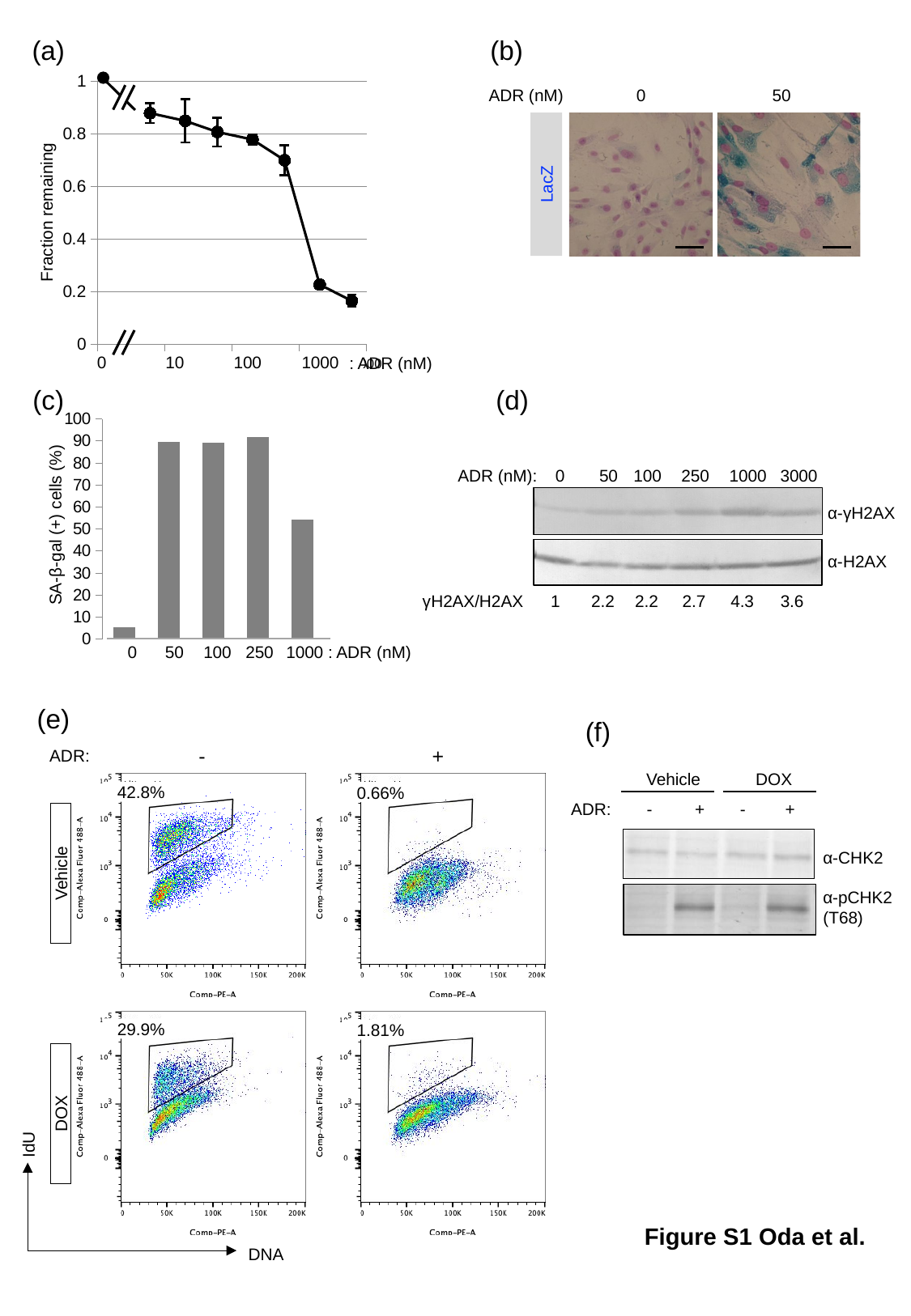

(a)
(b)
### Chart
| Category | Y の値 1 |
|---|---|
Fraction remaining
0
10
100
1000
: ADR (nM)
ADR (nM)
0
50
LacZ
(c)
 (d)
### Chart
| Category | 系列 1 |
|---|---|
| 0 nM | 5.401844532 |
| 50 nM | 89.7515528 |
| 100 nM | 89.23357664 |
| 250 nM | 91.66666667 |
| 1000 nM | 54.38596491 |SA-β-gal (+) cells (%)
0
50
100
250
1000
: ADR (nM)
ADR (nM):
0
50
100
250
1000
3000
α-γH2AX
α-H2AX
γH2AX/H2AX
1
2.2
2.2
2.7
4.3
3.6
 (e)
 (f)
Vehicle
DOX
ADR:
-
+
-
+
α-CHK2
-
+
ADR:
42.8%
0.66%
Vehicle
29.9%
1.81%
DOX
IdU
DNA
α-pCHK2 (T68)
Figure S1 Oda et al.

## Slide 2
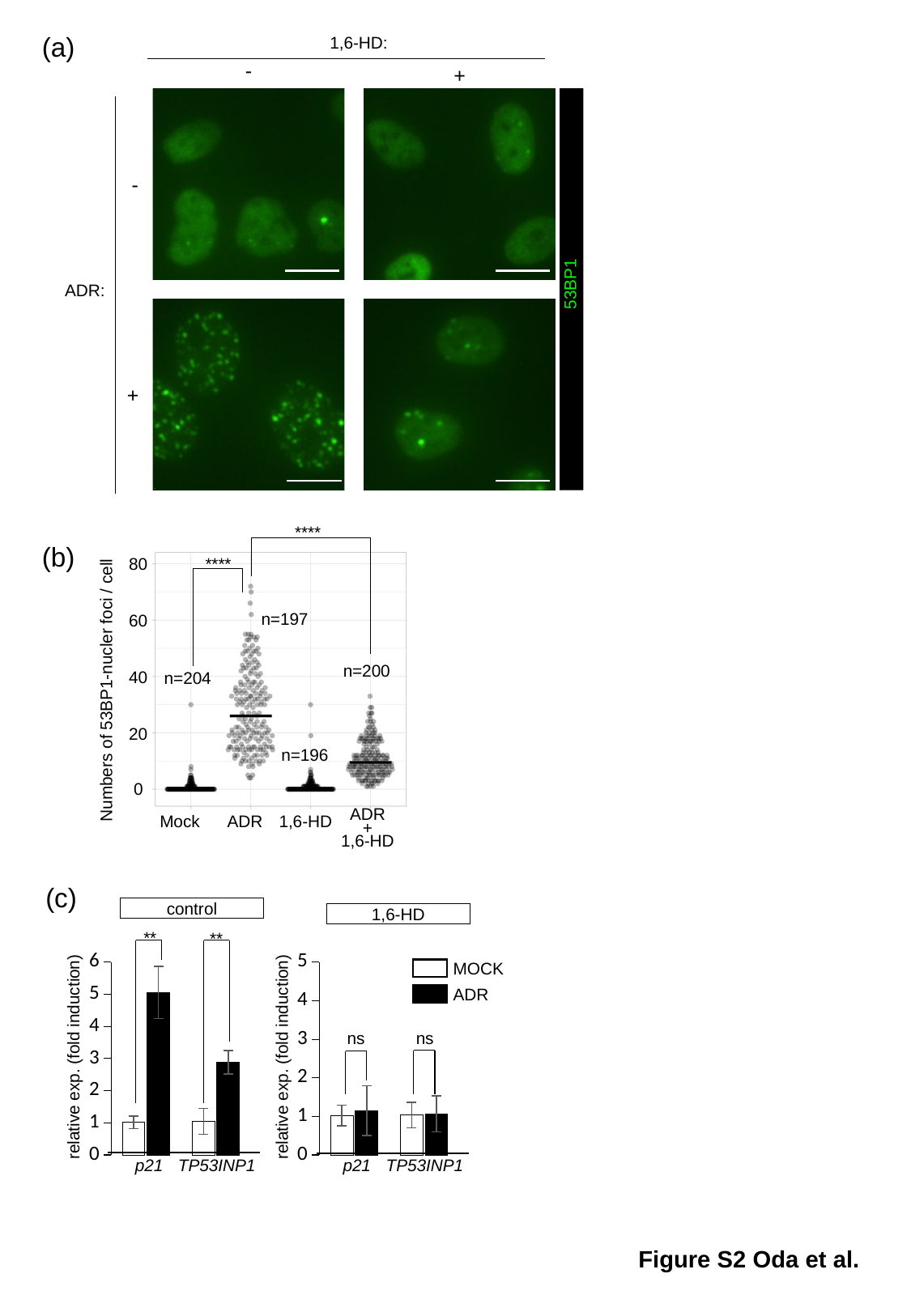

(a)
1,6-HD:
-
+
-
53BP1
ADR:
+
****
(b)
80
n=197
60
n=200
n=204
40
Numbers of 53BP1-nucler foci / cell
20
n=196
0
Mock
ADR
1,6-HD
ADR
+
1,6-HD
****
(c)
control
### Chart
| Category | Mock | ADR |
|---|---|---|
| p21 | 1.012539448143383 | 5.060062471622473 |
| TP53INP1 | 1.0460581796030806 | 2.8859572902240695 |relative exp. (fold induction)
p21
TP53INP1
**
**
1,6-HD
### Chart
| Category | Mock | ADR |
|---|---|---|
| p21 | 1.0235084850906933 | 1.1511802853871538 |
| TP53INP1 | 1.0346146249615507 | 1.0603987136822501 |relative exp. (fold induction)
p21
TP53INP1
MOCK
ADR
ns
ns
Figure S2 Oda et al.

## Slide 3
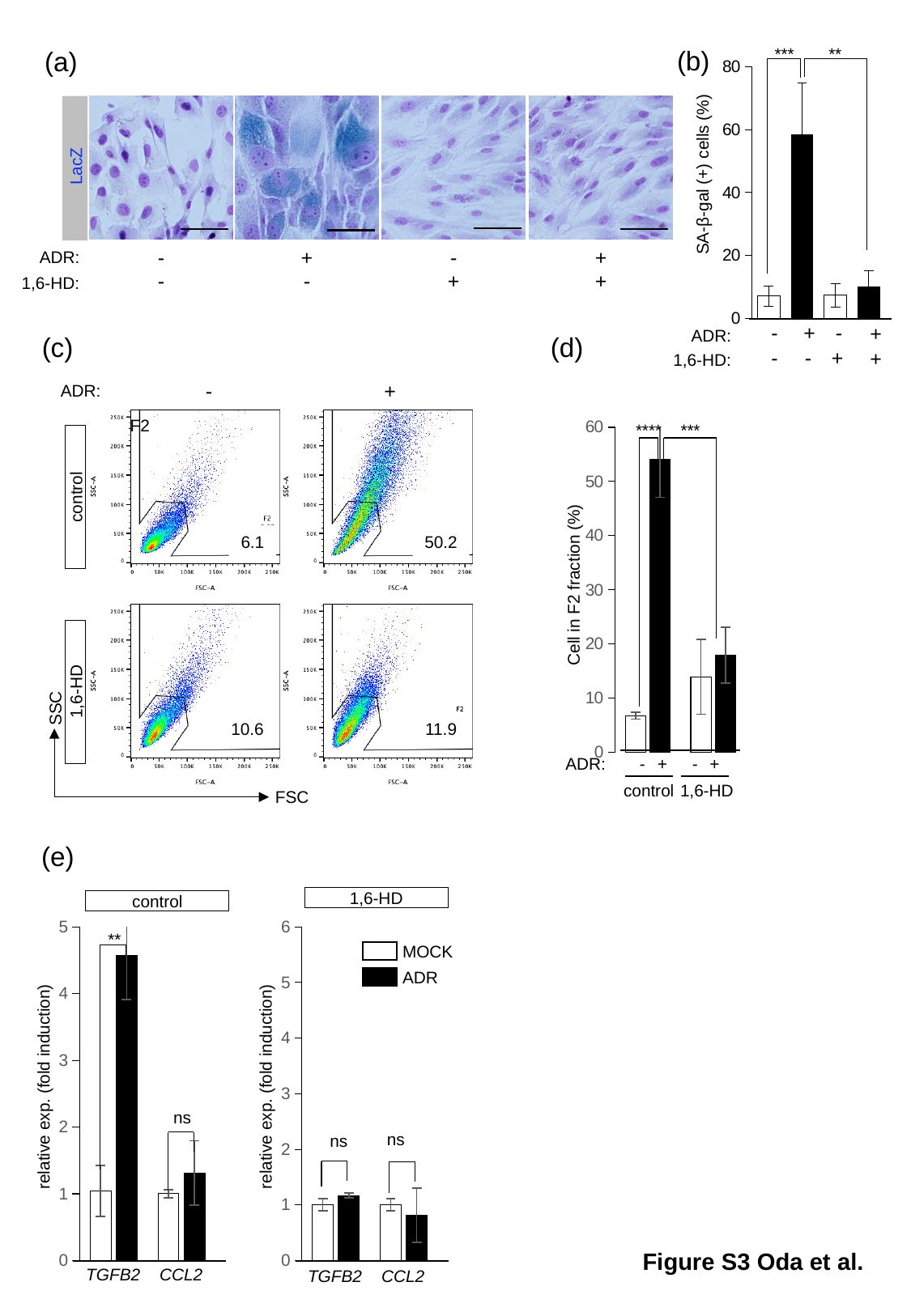

***
**
(b)
### Chart
| Category | 系列 1 |
|---|---|
| Control | 7.034517304280435 |
| ADR | 58.373037192888084 |
| 1,6-HD | 7.2981628965332614 |
| 1,6-HD + ADR | 10.205767479116897 |SA-β-gal (+) cells (%)
-
+
-
+
ADR:
-
-
+
+
1,6-HD:
(a)
LacZ
ADR:
-
+
-
+
-
-
+
+
1,6-HD:
(c)
(d)
-
+
ADR:
F2
control
6.1
50.2
1,6-HD
SSC
10.6
11.9
FSC
### Chart
| Category | MOCK | ADR |
|---|---|---|
| control | 6.7725 | 54.125 |
| 1,6-HD | 13.95 | 17.925 |Cell in F2 fraction (%)
ADR:
-
+
-
+
control
1,6-HD
****
***
(e)
1,6-HD
### Chart
| Category | MOCK | ADR |
|---|---|---|
| TGFB2 | 1.0040525497638007 | 1.170649231648629 |
| CCL2 | 1.004013713613855 | 0.813367448069366 |MOCK
ADR
relative exp. (fold induction)
TGFB2
CCL2
ns
ns
control
### Chart
| Category | MOCK | ADR |
|---|---|---|
| TGFB2 | 1.041988351743375 | 4.568178863063839 |
| CCL2 | 1.001157888988711 | 1.3142175791983925 |relative exp. (fold induction)
TGFB2
CCL2
**
ns
Figure S3 Oda et al.

## Slide 4
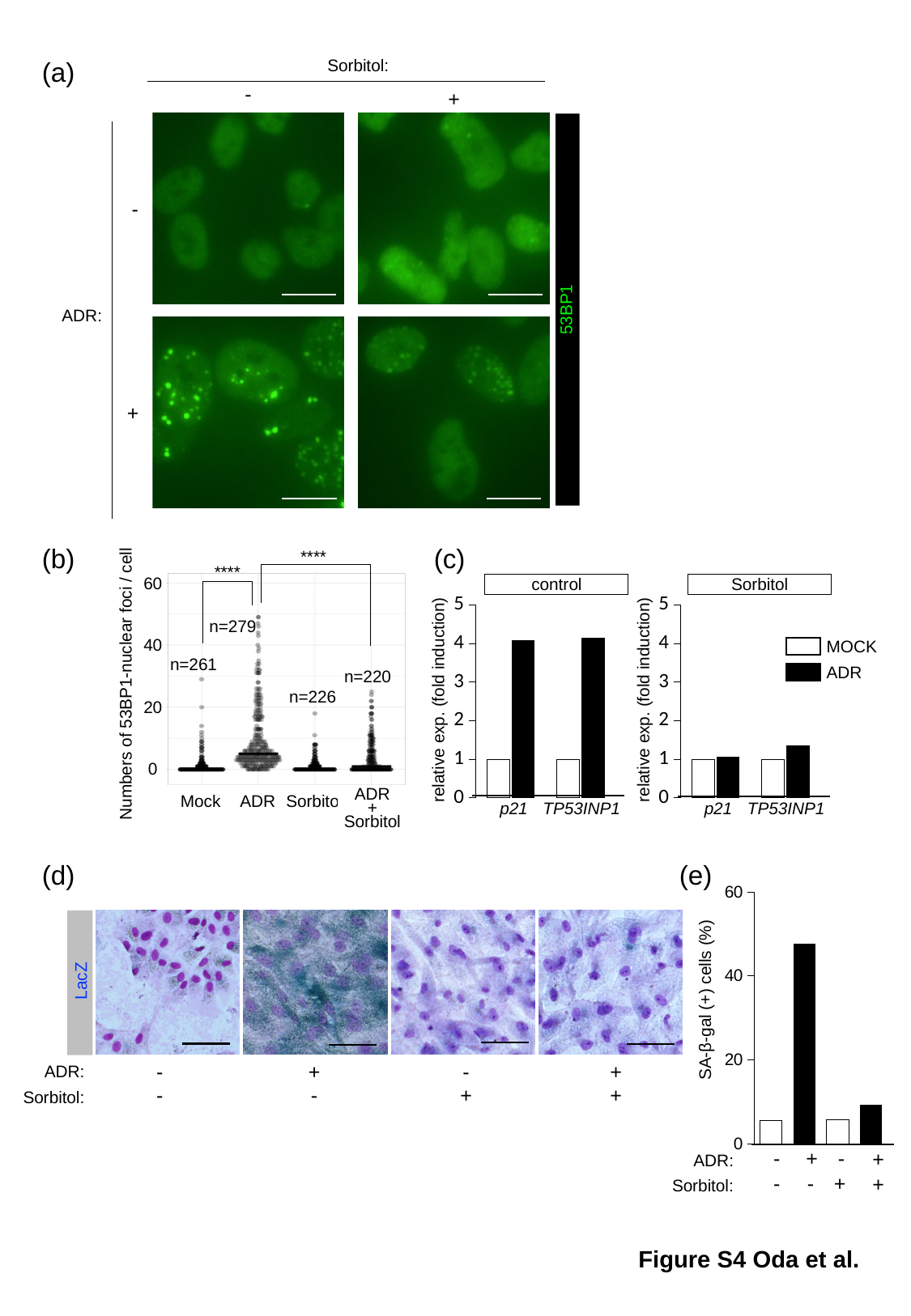

Sorbitol:
-
+
-
53BP1
ADR:
+
(a)
60
n=279
40
n=261
n=220
Numbers of 53BP1-nuclear foci / cell
n=226
20
0
Mock
ADR
Sorbitol
ADR
+
Sorbitol
****
****
(b)
(c)
### Chart
| Category | Mock | ADR |
|---|---|---|
| p21 | 1.0000000002553509 | 4.07401822944568 |
| TP53INP1 | 1.000000000337514 | 4.14276067364544 |relative exp. (fold induction)
p21
TP53INP1
control
### Chart
| Category | Mock | ADR |
|---|---|---|
| p21 | 0.9999999996225503 | 1.0522586205758322 |
| TP53INP1 | 1.000000000055671 | 1.3437994195014809 |relative exp. (fold induction)
p21
TP53INP1
Sorbitol
MOCK
ADR
(d)
(e)
### Chart
| Category | 系列 1 |
|---|---|
| siLacZ | 5.599500982109678 |
| siLacZ ADR | 47.72689853879399 |
| siRNF168 | 5.645159937122248 |
| siRNF168 ADR | 9.39199549451348 |SA-β-gal (+) cells (%)
-
+
-
+
ADR:
-
-
+
+
Sorbitol:
LacZ
ADR:
-
+
-
+
Sorbitol:
-
-
+
+
Figure S4 Oda et al.

## Slide 5
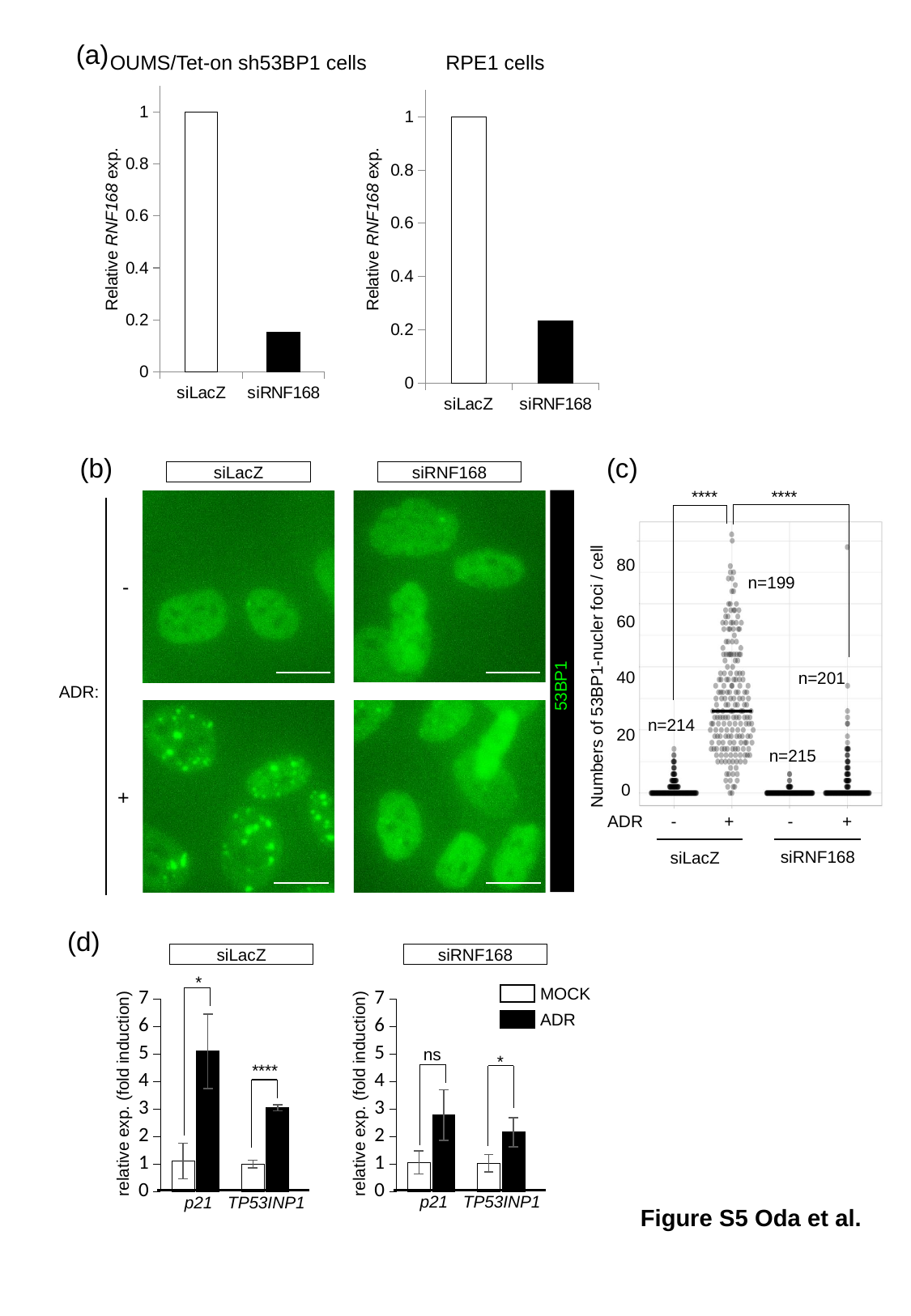

(a)
OUMS/Tet-on sh53BP1 cells
### Chart
| Category | RNF168 |
|---|---|
| siLacZ | 1.001015128693135 |
| siRNF168 | 0.15299007356768 |Relative RNF168 exp.
RPE1 cells
### Chart
| Category | RNF168 |
|---|---|
| siLacZ | 1.0004864981182124 |
| siRNF168 | 0.2335472282836745 |Relative RNF168 exp.
(b)
(c)
siLacZ
siRNF168
-
53BP1
ADR:
+
****
****
80
n=199
60
Numbers of 53BP1-nucler foci / cell
n=201
40
n=214
20
n=215
0
ADR
-
+
-
+
siRNF168
siLacZ
(d)
siLacZ
### Chart
| Category | Mock | ADR |
|---|---|---|
| p21 | 1.1162777029443502 | 5.101352959763943 |
| TP53INP1 | 1.0060292619706233 | 3.046873384049663 |relative exp. (fold induction)
p21
TP53INP1
*
****
siRNF168
MOCK
ADR
### Chart
| Category | Mock | ADR |
|---|---|---|
| p21 | 1.060518938752951 | 2.7870995306332174 |
| TP53INP1 | 1.0340761874693947 | 2.159908632895109 |relative exp. (fold induction)
p21
TP53INP1
ns
*
Figure S5 Oda et al.

## Slide 6
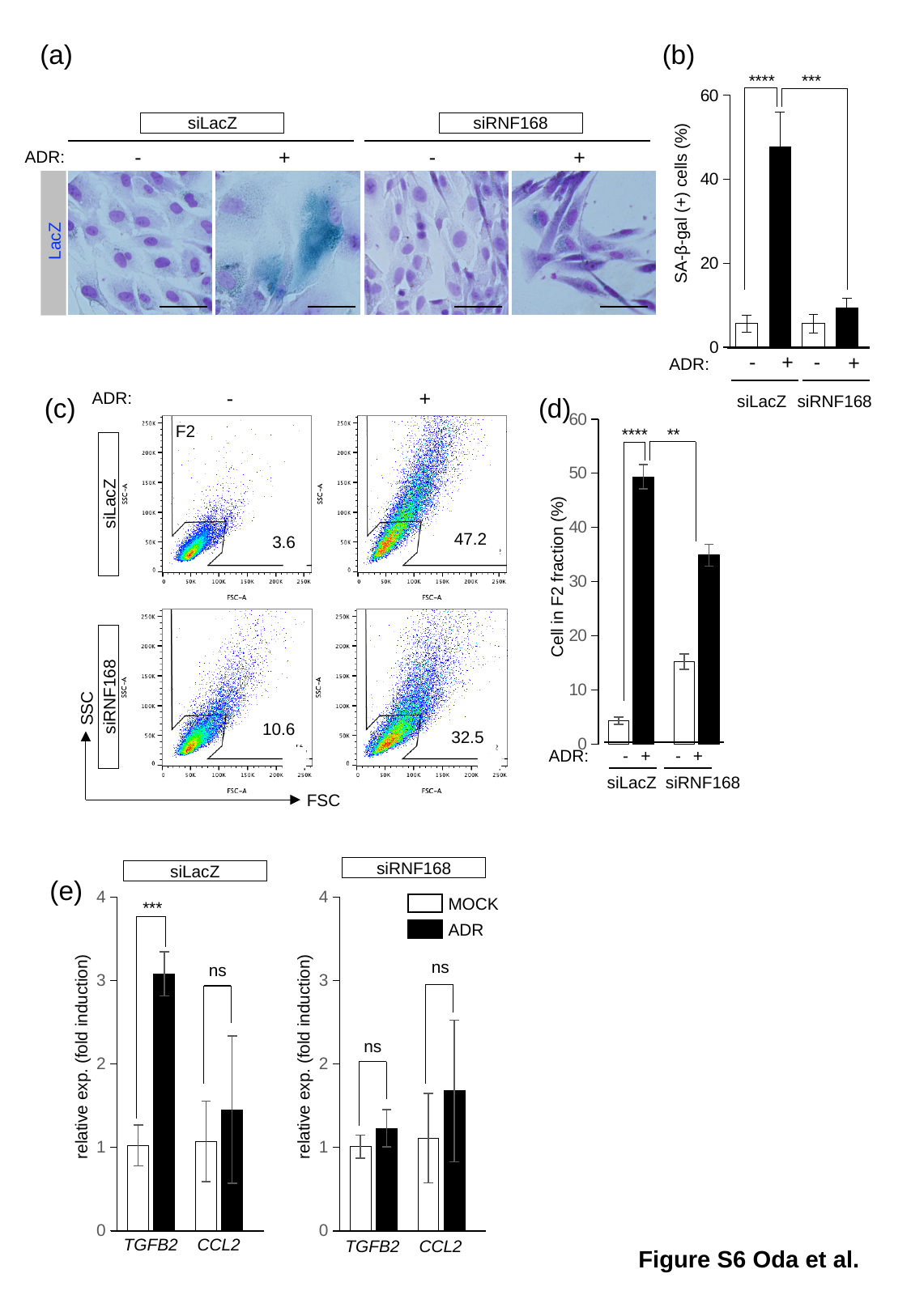

(a)
(b)
****
***
### Chart
| Category | 系列 1 |
|---|---|
| Control | 5.599500982109678 |
| ADR | 47.72689853879399 |
| 1,6-HD | 5.645159937122248 |
| 1,6-HD + ADR | 9.39199549451348 |SA-β-gal (+) cells (%)
-
+
-
+
ADR:
siLacZ
siRNF168
siLacZ
siRNF168
ADR:
-
+
-
+
LacZ
-
+
ADR:
F2
siLacZ
47.2
3.6
siRNF168
SSC
10.6
32.5
FSC
(c)
(d)
### Chart
| Category | MOCK | ADR |
|---|---|---|
| siLacZ | 4.343333333333333 | 49.366666666666674 |
| siRNF168 | 15.233333333333334 | 34.9 |Cell in F2 fraction (%)
ADR:
-
+
-
+
siLacZ
siRNF168
****
**
siRNF168
### Chart
| Category | MOCK | ADR |
|---|---|---|
| TGFB2 | 1.006087371134073 | 1.2255598713158091 |
| CCL2 | 1.109093606248644 | 1.6743945989540752 |MOCK
ADR
relative exp. (fold induction)
TGFB2
CCL2
ns
ns
siLacZ
### Chart
| Category | MOCK | ADR |
|---|---|---|
| TGFB2 | 1.0214509285376805 | 3.077495090262372 |
| CCL2 | 1.0697914188330402 | 1.4506048655885617 |relative exp. (fold induction)
TGFB2
CCL2
***
ns
(e)
Figure S6 Oda et al.

## Slide 7
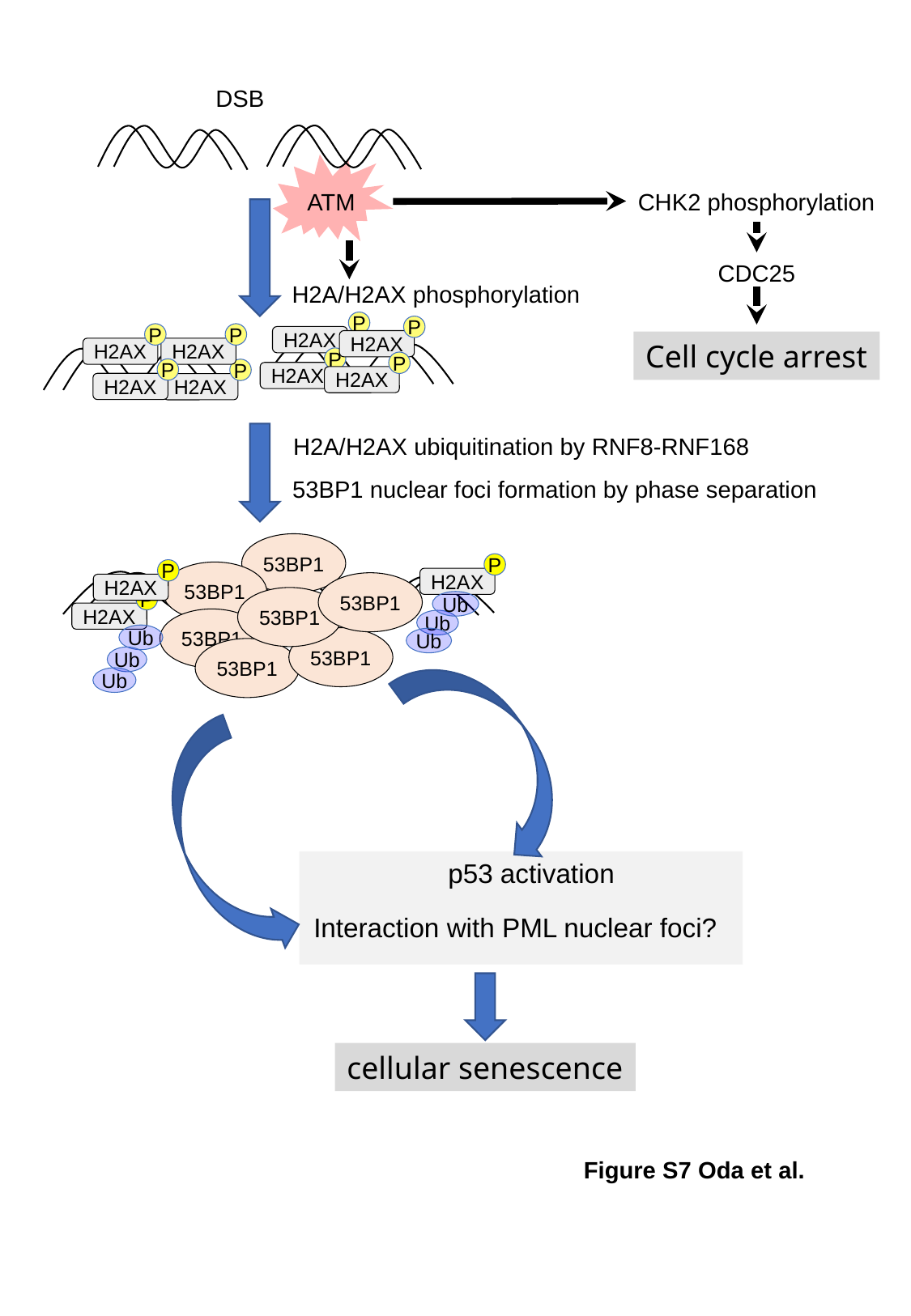

DSB
CHK2 phosphorylation
ATM
CDC25
H2A/H2AX phosphorylation
P
H2AX
P
H2AX
P
H2AX
P
H2AX
P
H2AX
P
H2AX
P
H2AX
P
H2AX
Cell cycle arrest
H2A/H2AX ubiquitination by RNF8-RNF168
53BP1 nuclear foci formation by phase separation
53BP1
P
P
53BP1
H2AX
53BP1
H2AX
53BP1
P
Ub
H2AX
53BP1
Ub
Ub
53BP1
Ub
53BP1
Ub
Ub
p53 activation
Interaction with PML nuclear foci?
cellular senescence
Figure S7 Oda et al.
